# Culture-Expanded Human Invariant Natural Killer T Cells Suppress T-Cell Alloreactivity and Eradicate Leukemia

**DOI:** 10.3389/fimmu.2018.01817

**Published:** 2018-08-06

**Authors:** Hannes Schmid, Corina Schneidawind, Simona Jahnke, Felix Kettemann, Kathy-Ann Secker, Silke Duerr-Stoerzer, Hildegard Keppeler, Lothar Kanz, Paul B. Savage, Dominik Schneidawind

**Affiliations:** ^1^Department of Medicine II, University Hospital Tübingen, Eberhard Karls University, Tübingen, Germany; ^2^Department of Chemistry and Biochemistry, Brigham Young University, Provo, UT, United States

**Keywords:** cytotherapy, graft-versus-host disease, graft-versus-leukemia, hematopoietic cell transplantation, invariant natural killer T cells

## Abstract

Graft-versus-host disease (GVHD) is a major cause of significant morbidity and mortality after allogeneic hematopoietic cell transplantation (HCT). Invariant natural killer T (iNKT) cells are potent regulators of immune responses, protect from lethal GVHD, and promote graft-versus-leukemia effects in murine studies. Since iNKT cells constitute less than 0.5% of human peripheral blood mononuclear cells (PBMCs), *in vitro* expansion with their glycolipid ligands is required before they can be used for cytotherapy and experimental purposes. Three weeks of cell culture and autologous restimulation with either KRN7000, PBS44, or PBS57 resulted in a robust proliferation of iNKT cells from human PBMCs. Next, iNKT cells were sorted to a purity higher than 90% being crucial for further experimental and clinical applications. These iNKT cells significantly decreased activation and proliferation of allogeneic CD3+ T lymphocytes. In addition, leukemia cell lines and primary leukemia cells were efficiently lysed by culture-expanded iNKT cells. Importantly, culture-expanded donor iNKT cells promoted robust antileukemia activity against HLA-matched allogeneic patient leukemia cells. Our data indicate that the adoptive transfer of culture-expanded iNKT cells could be a powerful cytotherapeutic approach to induce immune tolerance and prevent leukemia relapse after allogeneic HCT in humans.

## Introduction

Invariant natural killer T (iNKT) cells constitute a small subset of T lymphocytes being defined by the expression of a semi-invariant T-cell receptor in both humans (TCRα Vα24-Jα18) and mice (TCRα Vα14-Jα18). Their functional hallmark is the instant release of immunoregulatory cytokines upon activation through glycolipids presented by the MHC-I-like molecule CD1d.

Recent preclinical and clinical studies highlight their capability to induce immune tolerance in the context of allogeneic hematopoietic cell transplantation (HCT). Graft-versus-host disease (GVHD) is a serious and sometimes fatal complication following allogeneic HCT caused by alloreactive donor T cells leading to inflammation of GVHD target organs like the gut, skin, and liver (1, 2). Adoptively transferred iNKT cells successfully prevent acute and chronic GVHD in murine models of allogeneic HCT while promoting beneficial graft-versus-leukemia (GVL) effects (3–5).

Moreover, it has been shown in humans and mice that minimal intensity conditioning with total lymphoid irradiation and antithymocyte globulin prior to allogeneic HCT leads to a preferential expansion of host iNKT cells that protect from GVHD and allow for successful combined HCT and solid organ transplantation (6–8). Therefore, adoptive transfer strategies would enable us to use iNKT cells independent of conditioning regimens. There is also a convincing body of clinical evidence that iNKT cells induce immune tolerance after allogeneic HCT in humans (9–11).

Consequently, adoptive transfer strategies of iNKT cells are urgently needed to utilize their tolerogenic and cytotoxic properties in the clinic. In this study, we investigated iNKT-cell expansion strategies using different glycolipids and tested the ability of culture-expanded human iNKT cells to control alloreactive T cells and kill leukemia cells.

## Materials and Methods

### Research Subjects

Human buffy coats from healthy volunteers were obtained from the Center of Clinical Transfusion Medicine Tübingen. Primary leukemia cells were cryopreserved from untreated patients after informed consent was obtained. The study was approved by our institutional review board to be in accordance with the ethical standards and with the Helsinki Declaration of 1975, as revised in 2013 (IRB approvals 483/2015BO2 and 137/2017BO2).

### iNKT-Cell Expansion

iNKT cells were expanded from human peripheral blood mononuclear cells (PBMCs) in iNKT-cell culture medium consisting of RPMI 1640 GlutaMAX™ Medium (ThermoFisher Scientific, Waltham, MA, USA), 10% FBS (fetal bovine serum, Biochrom, Berlin, Germany), 100 IU/ml penicillin–streptomycin (Lonza, Basel, Switzerland), 5.5 µM 2-mercaptoethanol (Roth, Karlsruhe, Germany), 0.1 mM non-essential amino acids (NEAA) (Gibco, Grand Island, New York, NY, USA), 10 mM HEPES (Gibco, Grand Island, New York, NY, USA), and 1 mM sodium pyruvate (Gibco, Grand Island, New York, NY, USA). 2 × 10^6^ PBMCs/ml that contained >0.05% iNKT cells were co-incubated with 100 ng/ml KRN7000 (Sigma-Aldrich, St. Louis, MO, USA), PBS44, or PBS57 and 100 IU/ml recombinant human interleukin 2 (rhIL-2, Novartis, Basel, Switzerland) in a 24-well culture plate. KRN7000 was developed and based on the structures of glycolipids discovered in the marine sponge *Agelas mauritianus* (12). PBS57 was developed as an alternate α-galactosylceramide to KRN7000, and in some assays generated iNKT-cell responses at lower concentrations than KRN7000 (13). Both PBS44 and PBS57 contain an unsaturated acyl chain, which may improve solubility and loading into CD1d. After 7 and 14 days, 1 × 10^6^ cultured cells were re-stimulated with 2 × 10^6^ irradiated (30 Gy, cesium-137 irradiator Gammacell 1000, Atomic Energy of Canada Limited, Chalk River, Canada) and glycolipid-pulsed autologous PBMCs (responder to feeder ratio 1:2) together with rhIL-2 (100 IU/ml) and the respective glycolipid (100 ng/ml) in a 12-well (second week) and 6-well (third week) culture plate. To generate glycolipid-pulsed autologous PBMCs, cells were co-incubated with 100 ng/ml of the respective glycolipid antigen at 37°C for 4 h prior to autologous restimulation. After a total of 21 days, cell culture was completed.

### Flow Cytometry

PBS57-loaded and unloaded human CD1d tetramers were obtained from the National Institutes of Health Tetramer Core Facility (Atlanta, GA, USA). The following antibodies were purchased from BD Biosciences (Franklin Lakes, NJ, USA) or BioLegend (San Diego, CA, USA): anti-CD3 (HIT3a/OKT3), anti-CD4 (RPA-T4), anti-CD8 (HIT8a), anti-CD25 (BC96), anti-IFN-γ (4S.B3), anti-IL-4 (MP4-25D2), anti-IL-17 (BL168). Fluorescence minus one controls were used for proper gating. To stain dead cells, eBioscience Fixable Viability Dyes eFluor™ 506 and 780 (ThermoFisher Scientific, Waltham, MA, USA) were used. Data were acquired on a LSR Fortessa flow cytometer (BD Biosciences, Franklin Lakes, NJ, USA) and analyses were performed with FlowJo 10.2 (Tree Star, Ashland, OR, USA).

### Magnetic-Activated Cell Sorting (MACS)

Culture-expanded human iNKT cells were stained with PBS57-CD1d tetramer phycoerythrin (PE) and enriched with anti-PE MicroBeads (Miltenyi Biotec, Bergisch Gladbach, Germany). CD3+ T cells were isolated from human PBMCs with anti-CD3 MicroBeads (Miltenyi Biotec, Bergisch Gladbach, Germany). A QuadroMACS™ Separator (Miltenyi Biotec, Bergisch Gladbach, Germany) and LS Columns (Miltenyi Biotec, Bergisch Gladbach, Germany) were used according to the manufacturer’s instructions.

### Fluorescence-Activated Cell Sorting (FACS)

Culture-expanded human iNKT cells were stained with 4′,6-diamidino-2-phenylindole (DAPI, Merck, Darmstadt, Germany), CD3, CD4, CD8, and PBS57-CD1d tetramer and purified on a FACS Aria II cell sorter (BD Biosciences, Franklin Lakes, NJ, USA).

### Mixed Lymphocyte Reaction

To generate dendritic cells (DCs), plastic-adherent monocytes isolated from PBMCs were cultured for 6 days in RPMI 1640 GlutaMAX™ Medium (ThermoFisher Scientific, Waltham, MA, USA), 10% FBS (Biochrom, Berlin, Germany), 100 IU/ml penicillin–streptomycin (Lonza, Basel, Switzerland), 11.4 µM 2-mercaptoethanol (Roth, Karlsruhe, Germany), 0.1 mM NEAA (Gibco, Grand Island, New York, NY, USA), and 1 mM sodium pyruvate (Gibco, Grand Island, New York, NY, USA) supplemented with 50 ng/ml IL-4 and 100 ng/ml GM-CSF (Miltenyi Biotec, Bergisch Gladbach, Germany) every other day. Major-mismatched DCs (stimulators) were plated together with allogeneic CD3+ T cells (responders) at a 1:1 ratio and different doses of culture-expanded MACS or FACS purified donor iNKT cells. Cells were analyzed by flow cytometry for activation markers and proliferation after 1, 3 and 7 days, respectively.

### Cytokine Analysis

Cells were stimulated with 1× eBioscience Cell Stimulation Cocktail (ThermoFisher Scientific, Waltham, MA, USA) for 4 h at 37°C in iNKT-cell culture medium. After staining surface antigens, cells were fixed and permeabilized (ThermoFisher Scientific, Waltham, MA, USA) prior to staining of intracellular and intranuclear antigens. Stained cells were measured using a LSR Fortessa flow cytometer (BD Biosciences, Franklin Lakes, NJ, USA) and analyses were performed with FlowJo 10.2 (Tree Star, Ashland, OR, USA).

### Carboxyfluorescein Succinimidyl Ester (CFSE) Dilution Assay

CD3+ MACS purified T cells were resuspended in phosphate-buffered saline (PBS, Gibco, Grand Island, New York, NY, USA) and stained with CellTrace CFSE cell proliferation kit (BioLegend, San Diego, CA, USA) for 5 min at room temperature. Immediately after staining, cells were washed in pure FBS then two times in PBS supplemented with 5% FBS and finally resuspended in iNKT-cell culture medium. CFSE-labeled CD3+ T cells were tested in a MLR. The percentage of proliferating CD3+CD4+ and CD3+CD8+ T cells was determined by flow cytometric analysis.

### Leukemia Cell Lysis Assay

Culture-expanded iNKT cells were co-incubated at increasing ratios with 4 × 10^4^ Jurkat cells (Clone E6-1, ATCC, Manassas, VA, USA), MOLT-4 cells (ATCC, Manassas, VA, USA), or primary leukemia cells in iNKT-cell culture medium for 16 h at 37°C in a 96-well round bottom plate. Leukemia cell lysis through iNKT cells was measured on a LSR Fortessa flow cytometer (BD Biosciences, Franklin Lakes, NJ, USA) as described previously (14). To identify tumor cells, PBS57-CD1d tetramer+ iNKT cells were excluded. Only tumor cell samples with a purity ≥90% were used for these experiments. In certain experiments, iNKT cells were culture-expanded from donor cells and co-incubated with primary leukemia cells from patients that received HLA-matched hematopoietic stem cell grafts from their respective donors (10/10-antigen/allele matched unrelated donors). Supernatants were analyzed with a Legendplex Human CD8/NK Panel (BioLegend, San Diego, CA, USA) according to the manufacturer’s instructions.

### Statistical Analysis

Student’s *t* test and analysis of variance were used for statistical analysis of two and three groups, respectively. *P* < 0.05 was considered statistically significant. Data were analyzed with Prism 7.01 (GraphPad Software, La Jolla, CA, USA). All experiments were performed in triplicates and repeated independently at least three times.

## Results

### Comparable iNKT-Cell Expansion Induced by Glycolipids

**Figure 1:**
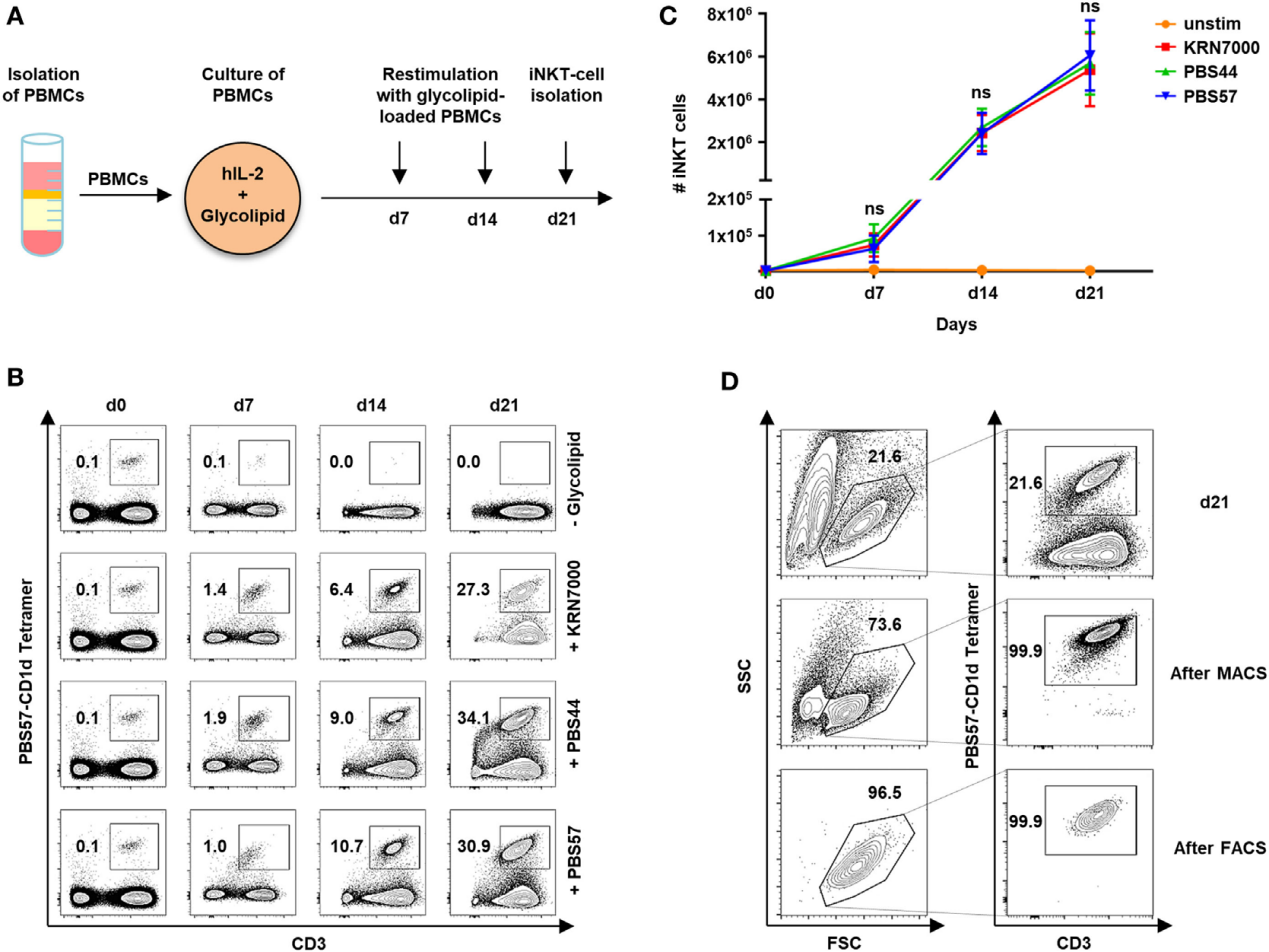
*Ex vivo* expansion and purification of human invariant natural killer T (iNKT) cells. **(A)** Scheme of iNKT-cell expansion from donor peripheral blood mononuclear cells (PBMCs) by glycolipid stimulation. iNKT cells were restimulated after 7 and 14 days with irradiated and glycolipid-pulsed autologous PBMCs. **(B)** Representative dot plots indicating percentage of iNKT cells cultured with KRN7000, PBS44, and PBS57 at days 0, 7, 14, and 21. All events were gated on live lymphocytes and iNKT cells were identified by expression of CD3 and staining with the PBS57-loaded CD1d tetramer. **(C)** Absolute iNKT-cell numbers during 21 days of expansion each starting from 2 × 10^6^ PBMCs. Results for all tested glycolipid analogs [KRN7000 (*n* = 9), PBS44 (*n* = 9), and PBS57 (*n* = 9)] are shown. Error bars indicate SEM. **(D)** Representative dot plots showing further purification of iNKT cells after 21 days of cell culture by magnetic-activated cell sorting (MACS) and fluorescence-activated cell sorting (FACS). FSC/SSC plots were gated on live lymphocytes and iNKT cells were identified by expression of CD3 and staining with the PBS57-loaded CD1d tetramer.

In our study cohort of 59 healthy volunteers, iNKT cells constitute 0.09% of PBMCs (range, 0.003–0.63). Although our previous studies showed that iNKT-cell/T-cell ratios of less than 1:20 are sufficient to protect from lethal GVHD in a murine model of allogeneic HCT (3), prior *ex vivo* expansion is required to obtain enough iNKT cells for cytotherapy in humans. We therefore, studied iNKT-cell expansion using the glycolipid KRN7000 and its derivatives PBS44 and PBS57. Figure 1A depicts the protocol of our 21-day cell culture with autologous restimulation. Cell numbers and immunophenotypes were assessed every 7 days by trypan blue vital stain and flow cytometry, respectively. Three weeks of cell culture resulted in a 7,130-fold (range, 1,852–16,886) expansion of iNKT cells with a purity of 30.4% (range, 8.4–62.1). However, stimulation with different glycolipids had no impact on iNKT-cell expansion capacity (Figures 1B,C). In addition, using the PBS57-loaded CD1d tetramer, iNKT cells could be purified to 95.7% (range, 78.4–99.9) by MACS or 94.2% (range, 78.7–100) by FACS following iNKT-cell culture (Figure 1D).

### Preferential Expansion of Tolerogenic CD4+ iNKT Cells

**Figure 2:**
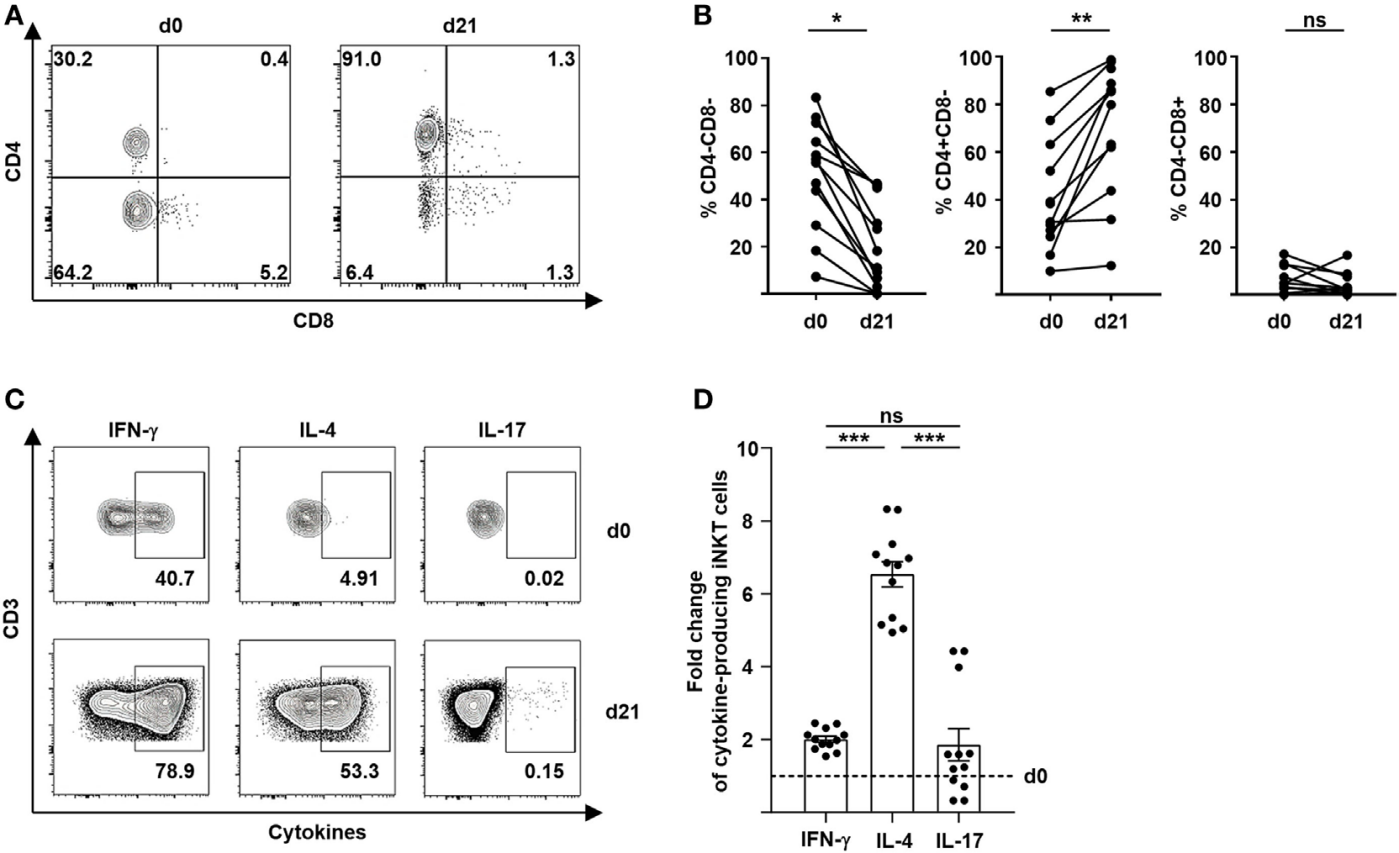
Preferential expansion of Th2-biased CD4+ invariant natural killer T (iNKT) cells. **(A)** Representative dot plots showing iNKT-cell subsets defined by the expression of CD4 and CD8 before and 21 days after cell culture. All events are gated on live iNKT cells. **(B)** Relative change of CD4−CD8− iNKT cells (*n* = 12), CD4+CD8− iNKT cells (*n* = 11), and CD4−CD8+ iNKT cells (*n* = 11) over 21 days of cell culture. **(C)** Representative dot plots showing Th1, Th2, and Th17 cytokine production of iNKT cells before and 21 days after cell culture. All events are gated on live iNKT cells. **(D)** Fold change of cytokine-producing iNKT cells at day 21 of cell culture compared to day 0 (dashed line) is shown for IFN-γ, IL-4, and IL-17 (*n* = 12). Error bars indicate SEM, *p < 0.05, **p < 0.01, ***p < 0.001.

The immunoregulatory properties of iNKT cells largely depend on the release of effector molecules and cytokines. It has been shown previously that a Th2 immune bias of T cells induced by CD4+ iNKT cells is critical for tolerance induction and prevention of GVHD (3, 4, 7, 8). We therefore, studied the immune polarization of culture-expanded iNKT-cell subsets after stimulation with different glycolipids. Remarkably, we observed a preferential expansion of CD4+ iNKT cells from 40% (range, 9.9–85.4) to 63% (range, 12.0–98.8) (Figures 2A,B). In contrast, percentage of CD8+ iNKT cells did not change significantly whereas CD4−CD8− iNKT cells showed a relative decline after 21 days of cell culture (Figures 2A,B). However, no relevant difference of preferential CD4+ iNKT-cell expansion was noted when KRN7000, PBS44, or PBS57 were used for iNKT-cell stimulation (Figure S1A in Supplementary Material). Subsequently, we performed intracellular cytokine staining of surrogate cytokines IFN-γ, IL-4, and IL-17 to evaluate cytokine profiles of culture-expanded iNKT cells (Figure 2C). All iNKT-cell subsets were functional as assessed by effective cytokine production after 21 days of cell culture. The choice of glycolipid for cell culture did not affect immune polarization of culture-expanded iNKT cells (Figure S1B in Supplementary Material). As already indicated by an increasing CD4+ iNKT-cell population, we observed a significant shift to Th2-biased iNKT cells when compared with unstimulated iNKT cells prior to iNKT-cell expansion (Figure 2D). Detailed cytokine profiles of iNKT-cell subsets before and after cell culture are shown in Figure S1C in Supplementary Material. Our data indicate a preferential expansion of highly tolerogenic iNKT cells irrespective of the glycolipid used for cell culture.

### Potent Inhibition of T-Cell Activation and Expansion by Culture-Expanded iNKT Cells

**Figure 3:**
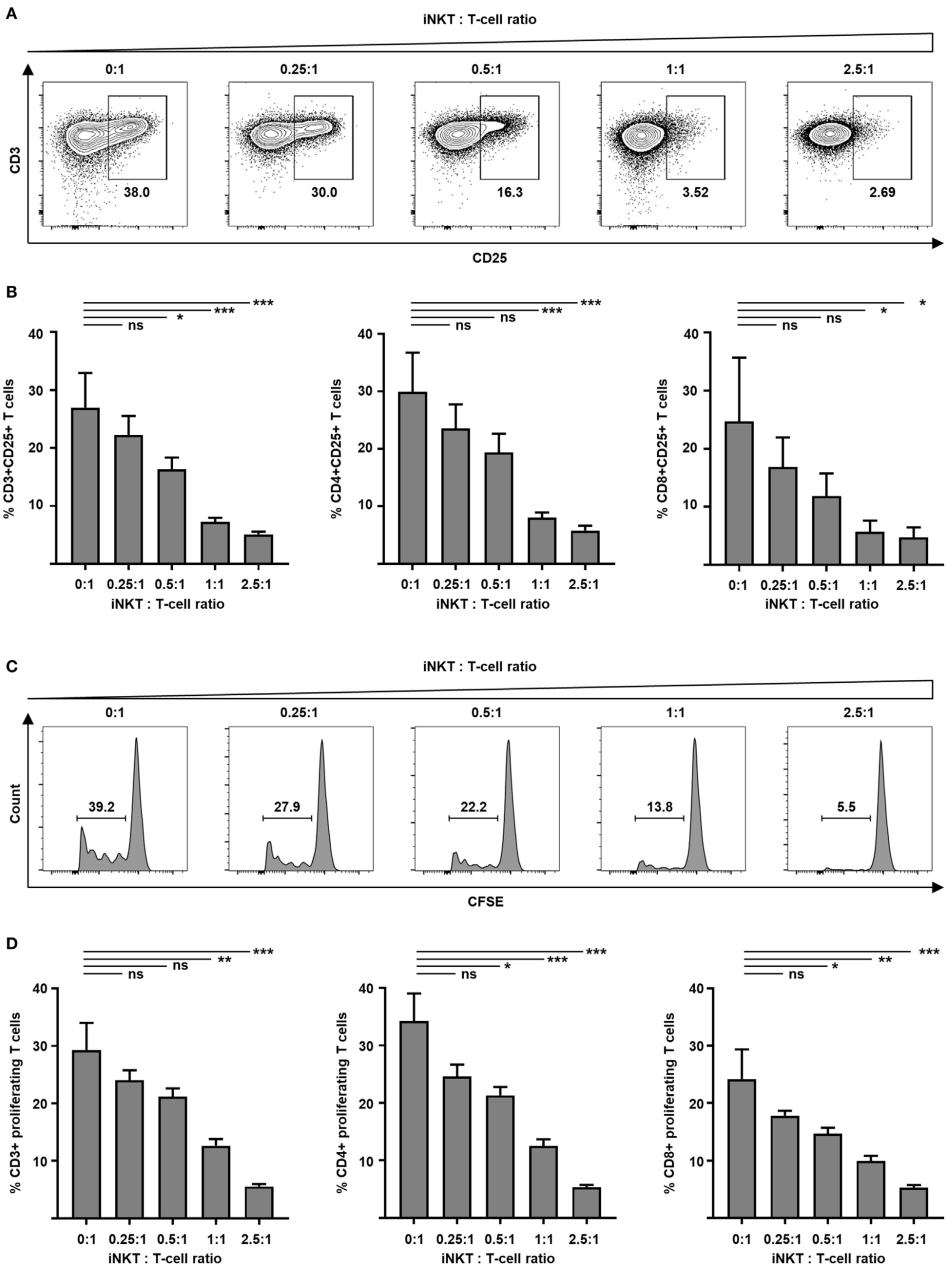
Suppression of allogeneic T-cell activation and proliferation by culture-expanded invariant natural killer T (iNKT) cells. Function of culture-expanded iNKT cells was tested in a MLR. Dendritic cells (recipient) and allogeneic major-mismatched CD3+ T cells (donor) were used as stimulators and responders at a 1:1 ratio, respectively. Increasing numbers of expanded iNKT cells from donor origin were added to the culture. **(A)** Representative dot plots of CD3+ responder T cells and **(B)** pooled data from four independent experiments of CD25 expression on live CD3+ responder T cells and CD4+ and CD8+ subsets at day + 3. Dot plots are gated on live lymphocytes and iNKT cells were excluded by prior CFSE staining. **(C)** Representative CFSE dilution histograms and **(D)** pooled data from three independent experiments of live allogeneic CD3+ T cells and CD4+ and CD8+ subsets at day + 7. Error bars indicate SEM, *p < 0.05, **p < 0.01, ***p < 0.001.

GVHD is mediated by alloreactive donor T cells that encounter antigen-presenting cells in secondary lymphoid organs of the host prior to destruction of target tissues. Preclinical animal studies demonstrate that adoptively transferred iNKT cells inhibit activation and proliferation of such alloreactive donor T cells. Therefore, we tested culture-expanded human iNKT cells for their capacity to control alloreactivity in a MLR. Donor T cells were analyzed by flow cytometry to determine cellular activation. We found a significantly decreased expression of early activation marker CD69 at day 1 (40.0 versus 70.8%, *p* = 0.03) and IL-2 receptor α-chain CD25 at day 3 (Figures 3A,B) on CD3+ T cells when culture-expanded iNKT cells were added. Comparable results were found for CD4+ and CD8+ T-cell subsets (Figure 3B). Fluorescence-activated cell sorted CD4+ iNKT cells and double negative (DN) iNKT cells (Figure S4A in Supplementary Material) were both potent suppressors of T-cell activation (Figure S4B in Supplementary Material). To study proliferation, donor T cells were labeled with CFSE prior to the MLR. At day 7, CFSE-labeled CD3+, CD4+, and CD8+ T cells were analyzed by flow cytometry. The percentage of proliferating cells was decreased in a dose-dependent manner when culture-expanded iNKT cells were added to the MLR (Figures 3C,D). Using the KRN7000 analogs PBS44 and PBS57 for iNKT-cell expansion resulted in comparable suppression of donor T-cell activation and proliferation (Figure S2 in Supplementary Material). Our studies indicate control of alloimmunity through culture-expanded human iNKT cells.

### Efficient Lysis of Leukemia Cells by Culture-Expanded iNKT Cells

**Figure 4:**
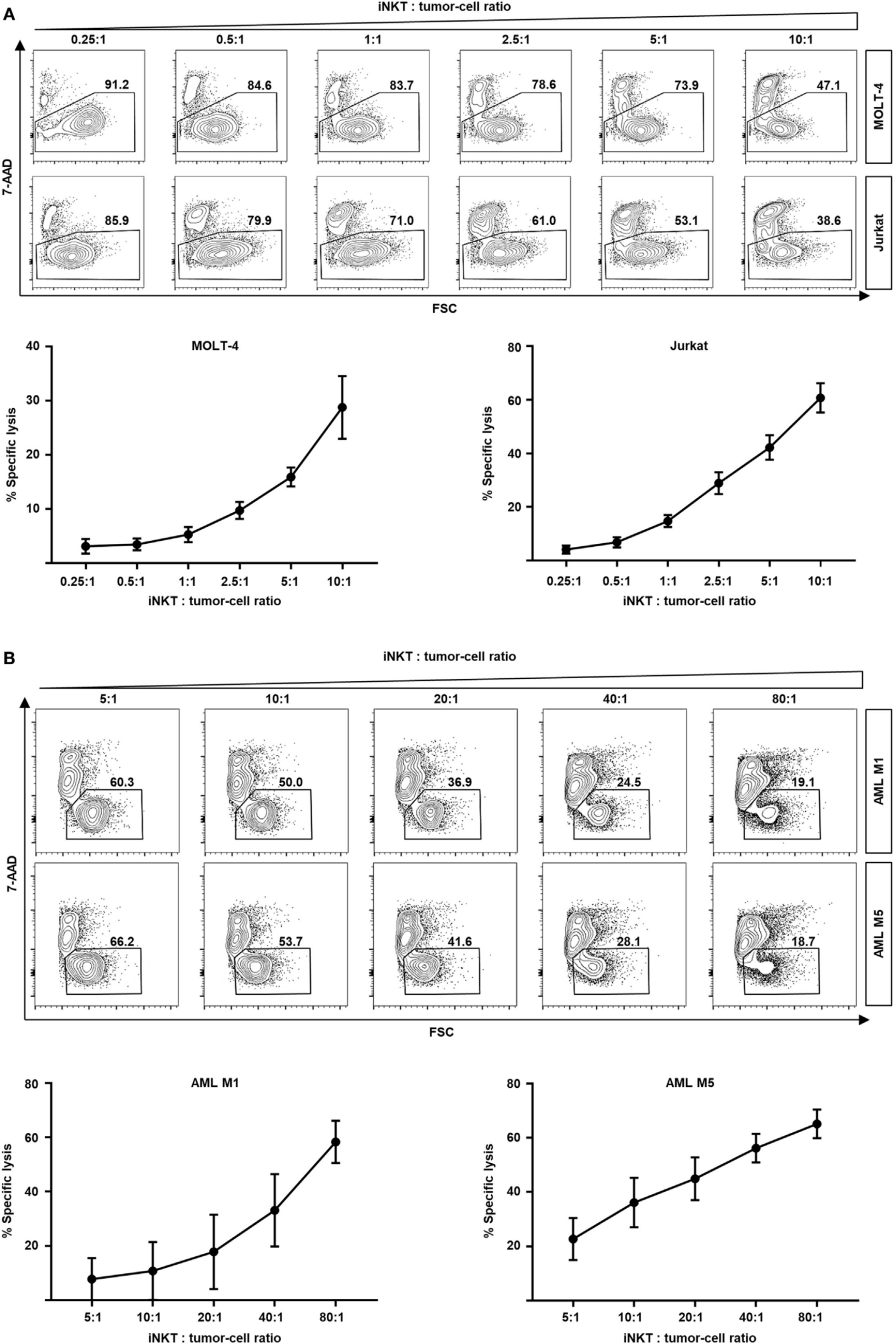

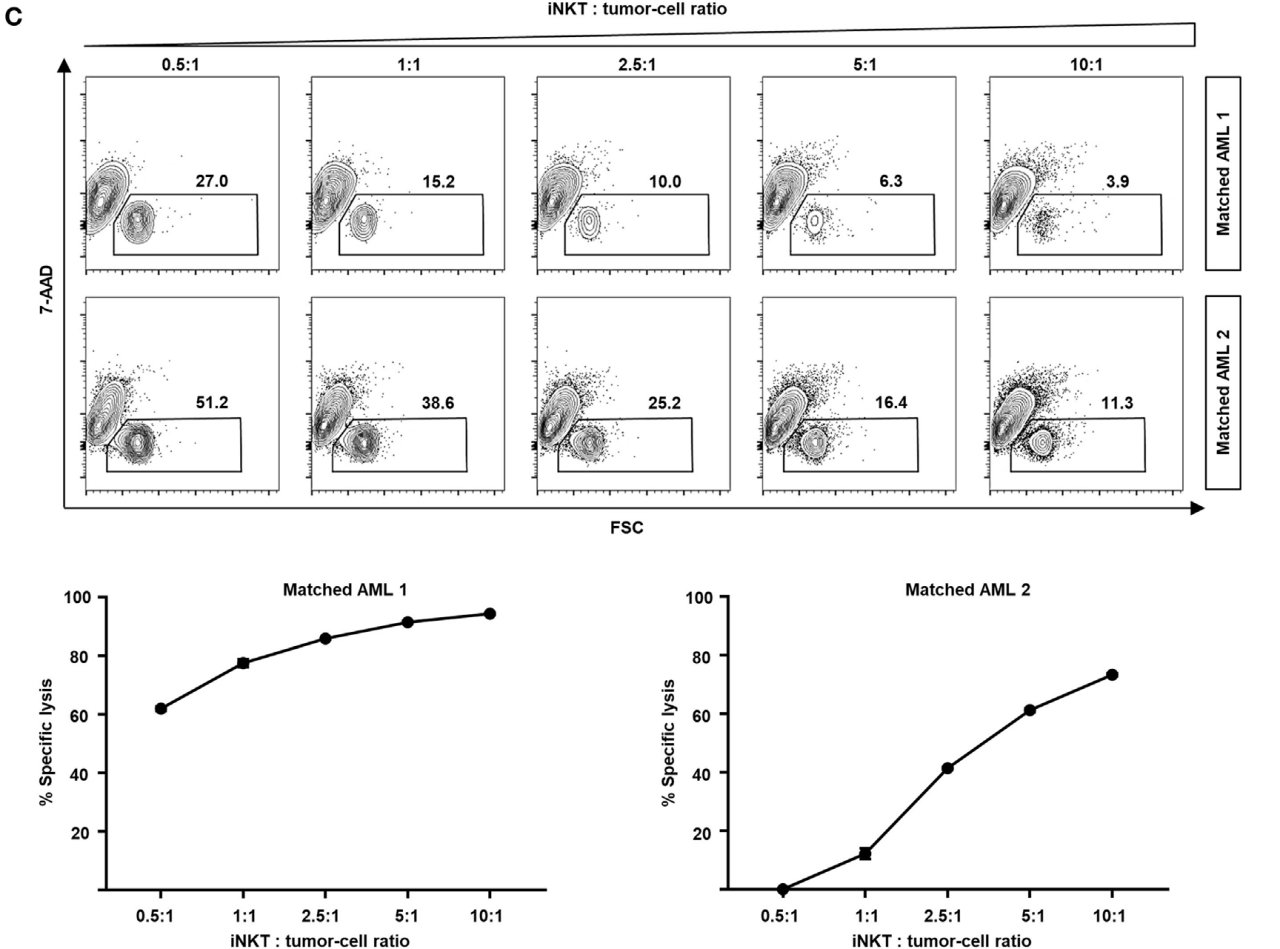
Dose-dependent kill of leukemia cell lines and patient leukemia cells by culture-expanded human invariant natural killer T (iNKT) cells. **(A)** Representative dot plots and specific lysis of MOLT-4 (92% 7-AAD- without iNKT cells) and Jurkat leukemia cells (87% 7-AAD- without iNKT cells) after co-incubation with culture-expanded iNKT cells. Each graph shows pooled data from four independent experiments with different culture-expanded iNKT cells. **(B)** Representative dot plots and specific lysis of AML M1 (88% 7-AAD- without iNKT cells) and AML M5 patient leukemia cells (89% 7-AAD- without iNKT cells) after co-incubation with culture-expanded HLA-mismatched iNKT cells. Each graph shows pooled data from four independent experiments with different culture-expanded iNKT cells tested against different patient AML cells. **(C)** Representative dot plots and specific lysis of patient leukemia cells (74 and 51% 7-AAD- without iNKT cells, respectively) after co-incubation with culture-expanded HLA-matched donor iNKT cells. Shown are two representative examples from three independent experiments. All events are gated on PBS57-loaded CD1d tetramer phycoerythrin negative cells to exclude iNKT cells. Error bars indicate SEM.

In addition of regulating immune responses by secretion of various cytokines, iNKT cells are capable of killing malignant cells through granzyme B and perforin or Fas–Fas ligand pathways (15–17). Moreover, rapid transactivation of other effector cells such as natural killer cells has been observed (18). Therefore, we studied the direct cytotoxic properties of culture-expanded human iNKT cells against various leukemia cell lines. Dose-dependent cell lysis of MOLT-4 and Jurkat cells was observed when co-cultured with iNKT cells for 16 h (Figure 4A). Comparable results were found for the KRN7000 analogs PBS44 and PBS57 (Figures S3A,B in Supplementary Material). Co-incubation of culture-expanded iNKT cells with Jurkat cells induced the release of perforin, granzyme A, and granzyme B (Figure S3C in Supplementary Material). In addition, fluorescence-activated cell sorted DN iNKT cells more efficiently killed Jurkat cells compared with CD4+ iNKT cells (Figure S4C in Supplementary Material). Furthermore, we investigated whether culture-expanded human iNKT cells are capable of lysing patient leukemia cells. We found an efficient dose-dependent killing of AML M1 and AML M5 cells when co-incubated with iNKT cells for 16 h (Figure 4B). Finally, we expanded donor iNKT cells and co-incubated them with primary leukemia cells from hitherto untreated patients that subsequently received the respective HLA-matched hematopoietic stem cell graft to simulate GVL activity *ex vivo*. We could detect potent dose-dependent antileukemia effects although iNKT cells and patient leukemia cells were HLA-matched (Figure 4C). In conclusion, our data indicate that culture-expanded human iNKT cells exert potent cytotoxic functions while controlling alloreactive immune responses.

## Discussion

Allogeneic HCT is the treatment of choice for advanced and high-risk hematopoietic malignancies. Optimized donor selection, tailored preparative conditioning regimes and advanced supportive care have significantly contributed to improved outcomes and enable long-term survival even in aged and comorbid patient populations. However, GVHD and relapse are still major reasons for significant morbidity and mortality after allogeneic HCT.

Donor-mediated inflammation of host tissues and efficient leukemia control are both sustained by alloreactive donor lymphocytes. Therefore, modulation of posttransplant immunity by immunosuppression or immunostimulation such as infusion of donor lymphocytes or check-point inhibitors increases the risk for relapse or GVHD, respectively (19–21). The induction of sustained immune tolerance while maintaining potent GVL effects is paramount to separate GVHD from leukemia control and further improve survival after allogeneic HCT.

Regulatory T lymphocytes turned out to be key elements in graft composition and consecutive modulation of posttransplant immune reconstitution. The highly tolerogenic minimal intensity conditioning regimen with total lymphoid irradiation and antithymocyte globulin depends on the persistence of host iNKT cells (6, 8). We could show previously that highly purified adoptively transferred iNKT cells prevent acute and chronic GVHD in a murine donor and third-party model of allogeneic HCT while promoting beneficial GVL effects (3, 4). Further clinical studies support our findings as iNKT-cell numbers in the graft and peripheral blood posttransplant were associated with a reduced incidence of GVHD and an improved GVHD-free and relapse-free survival (9–11).

However, it is mandatory to culture-expand iNKT cells to be able to use them for clinical applications since cell numbers in human peripheral blood are extremely low. In contrast to CD4+CD25+FoxP3+ regulatory T cells (Tregs), iNKT cells can be stimulated by using glycolipid ligands that engage the iNKTCR with both high affinity and specificity. Cytokines such as IL-2 are used to sustain iNKT-cell proliferation resulting in a robust expansion after 3 weeks of cell culture. KRN7000 and its analogs that were tested in this study promote the preferential expansion of IL-4 producing CD4+ iNKT cells. This subpopulation contains highly tolerogenic properties: Th2-biased iNKT cells turned out to be extremely potent in preventing autoimmune diseases such as experimental autoimmune encephalitis or GVHD (22, 23). Likewise, culture-expanded and purified human iNKT cells suppress activation and proliferation of alloreactive donor T cells in a MLR. Both steps are critical for GVHD induction in secondary lymphoid organs and subsequent target organ destruction and, therefore, support the results from our previous preclinical murine studies (3, 4, 24–26).

Meanwhile, donor lymphocyte-mediated GVL effects are critical to enable long-term disease-free survival after allogeneic HCT for leukemia treatment. We found previously that CD4+ iNKT cells exert robust antileukemia effects by themselves: BALB/c mice that were challenged with luciferase-expressing lymphoma cells showed a significantly improved survival after allogeneic HCT with T cell-depleted bone marrow cells and adoptive transfer of CD4+ iNKT cells both from C57BL/6 mice (3). Such findings correlate with several clinical studies showing that iNKT-cell counts after allogeneic HCT are associated with reduced relapse rates and improved survival (27, 28). iNKT cells are capable of inducing apoptosis of leukemia cells through the release of perforin and granzyme B or through Fas signaling (16, 17, 29). Moreover, iNKT cells create an immunological antileukemia environment by activating various innate or adaptive immune cells such as NK cells or T lymphocytes, respectively. Interestingly, injection of glycolipids into wild-type mice leads to a rapid iNKT cell-dependent activation of NK cells (18). Here, we show that culture-expanded iNKT cells exert dose-dependent antileukemia activity against cell lines and primary leukemia cells by releasing perforin and granzymes. In particular experiments, we were able to mimic the allogeneic transplant setting by challenging patient AML leukemia cells with their respective donor iNKT cells: iNKT cells that were expanded from cryopreserved donor PBMCs could efficiently lyse primary leukemia cells from their 10/10-HLA-matched unrelated recipient as well.

Therefore, culture-expanded iNKT cells from donor blood and subsequent infusion into transplant patients are a promising approach to prevent and treat relapse. This strategy might overcome the dose-limiting occurrence of GVHD after infusion of whole donor lymphocytes (30). Another approach to expand iNKT cells *in vivo* is the delivery of glycolipids on mature DCs as proposed by Chang et al. (31) However, insufficient iNKT-cell numbers of the host could constitute a drawback to induce clinically meaningful iNKT-cell responses. Therefore, our approach might be considered as an *ex vivo* transposition where glycolipid-pulsed DCs induce proliferation of donor iNKT cells from peripheral blood prior to adoptive transfer.

We also tested fluorescence-activated cell sorted CD4+ and DN iNKT-cell subpopulations to evaluate their individual immunoregulatory and cytolytic properties as the composition of iNKT-cell infusions might influence their clinical effectiveness. Due to low numbers of CD8+ iNKT cells after culture expansion, this subset could not be purified for further experiments. We found that DN iNKT cells lyse leukemia cells more efficiently suggesting an improved tumor control of DN iNKT cell-enriched cell products. However, DN and CD4+ iNKT cells showed a comparable efficacy in suppressing alloreactive T-cell responses. This observation is supported by Chaidos et al. who demonstrated that both CD4+ and CD4− iNKT-cell numbers of the graft are associated with a significantly reduced incidence of GVHD (10). Our data indicate that the cellular composition of iNKT-cell infusions need to be considered and adjusted when it comes to clinical translation.

In this study, we demonstrate the feasibility of iNKT-cell expansion from peripheral blood of healthy donors by using glycolipids that can be synthesized under good manufacturing practices (GMP) (32). This is important since all GMP requirements need to be fulfilled prior to clinical translation. These culture-expanded iNKT cells retain their tolerogenic capabilities and prevent activation and proliferation of alloreactive donor T cells while exerting potent cytotoxicity against cell lines and allogeneic patient leukemia cells. Therefore, adoptive transfer strategies using iNKT cells could induce immune tolerance while enhancing GVL effects following allogeneic HCT in humans.

## Ethics Statement

The study was approved by our institutional review board (Ethikkommission der Medizinischen Fakultät Tübingen) to be in accordance with the ethical standards and with the Helsinki Declaration of 1975, as revised in 2013 (IRB approvals 483/2015BO2 and 137/2017BO2).

## Author Contributions

HS and CS designed and performed experiments and analyzed data. SJ, FK, K-AS, SD-S, HK, LK, and PS performed experiments. PBS44 and PBS57 were synthesized by PS. DS designed experiments and provided overall guidance, HS, CS, and DS wrote the manuscript.

## Conflict of Interest Statement

The authors declare that the research was conducted in the absence of any commercial or financial relationships that could be construed as a potential conflict of interest.
