# Supplementary material for: Culture-Expanded Human Invariant Natural Killer T Cells Suppress T-Cell Alloreactivity and Eradicate Leukemia
Source: Front Immunol. 2018 Aug 6;9:1817. doi: 10.3389/fimmu.2018.01817 (PMC6088196; doi:10.3389/fimmu.2018.01817)
Supplement: Supplementary file 1 [file presentation_1.pdf]

A

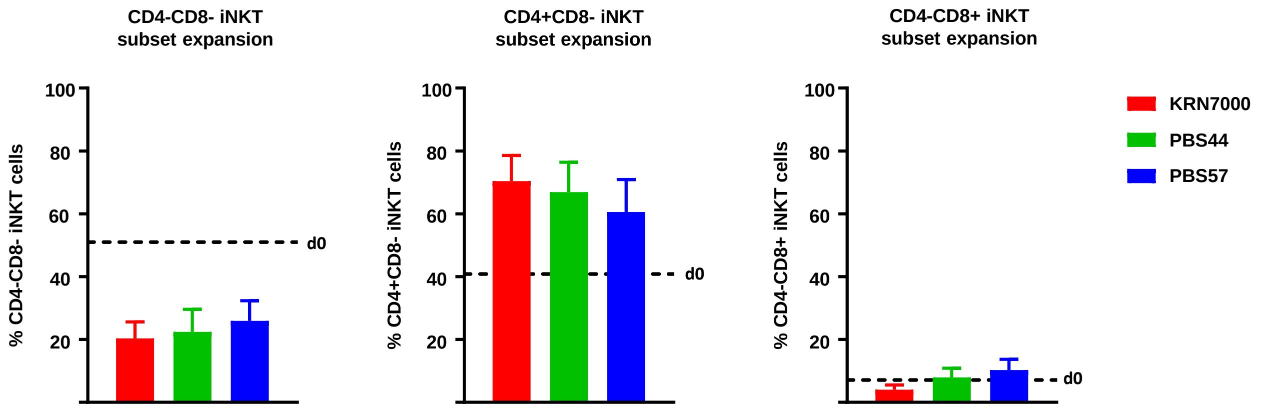

B

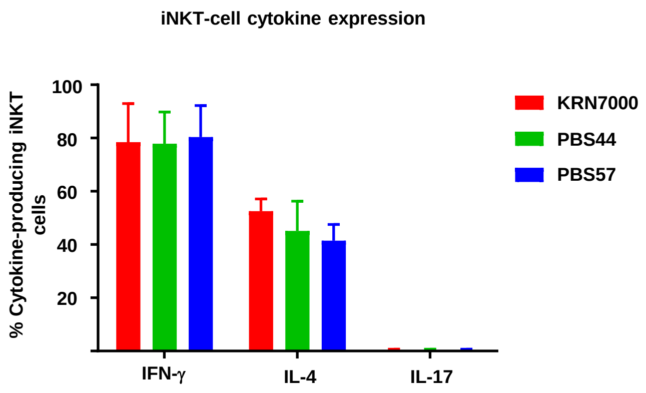

Schmid et al., Supplemental Figure 1

C

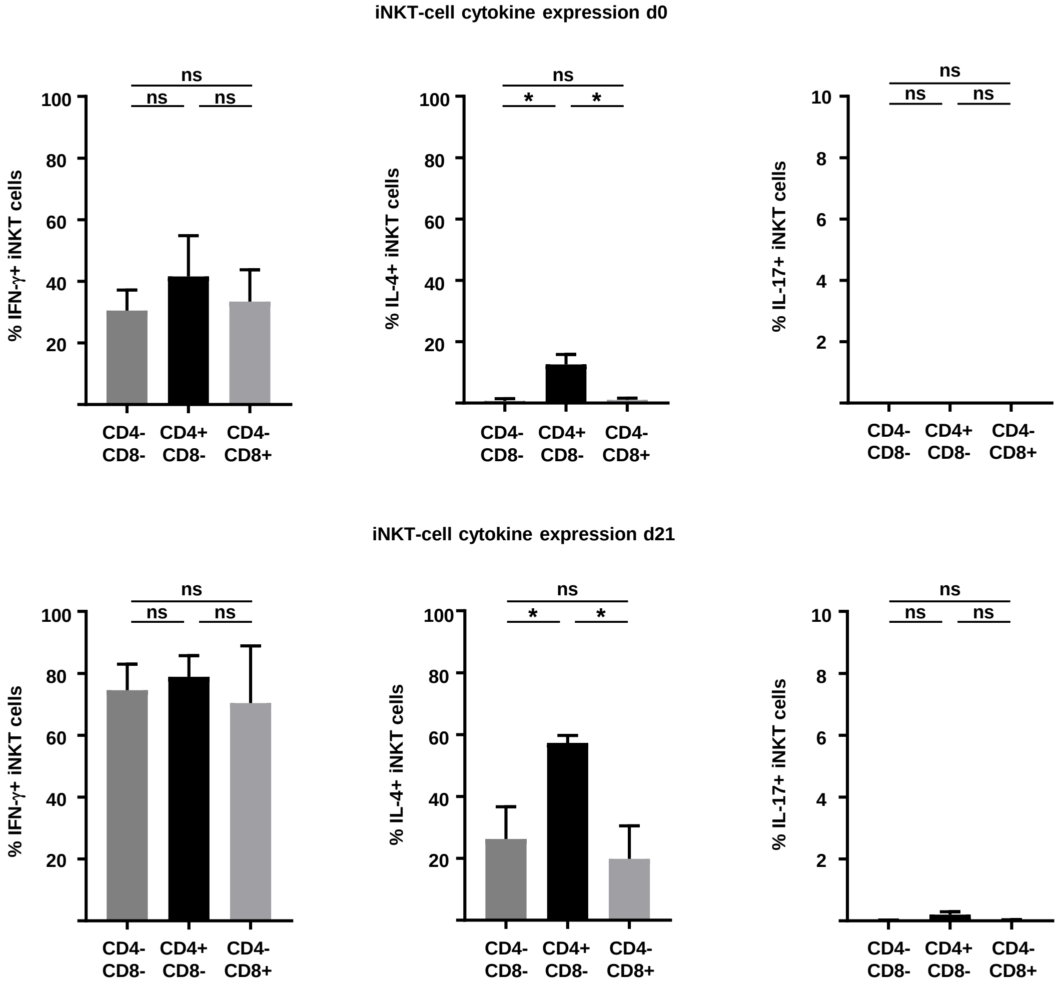

Supplemental Figure 1. Preferential expansion of Th2-biased CD4+ iNKT cells. (**A**) Percentage of iNKT-cell subsets after cell culture compared with the mean percentage of day 0 indicated by the dashed line. Pooled data from 12 independent samples. (**B**) Percentage of cytokine-producing iNKT cells after 21 days of cell culture with either KRN7000, PBS44 or PBS57. Pooled data from four independent samples. (**C**) Percentage of cytokine-producing iNKT-cell subsets (CD4-CD8-, CD4+CD8-, CD4-CD8+) before and after 21 days of cell culture. Pooled data from three independent samples. Error bars indicate standard error of the mean.

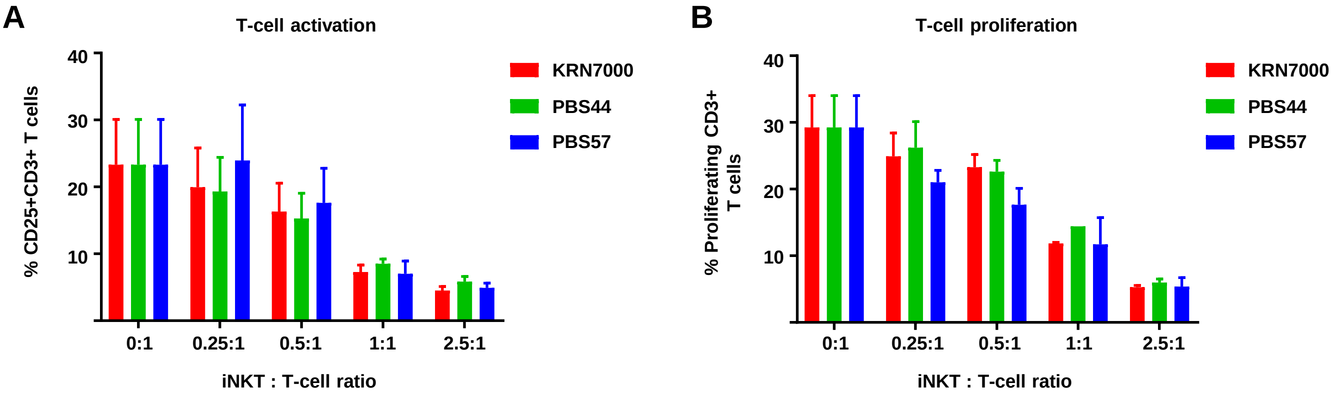

Supplemental Figure 2. Inhibition of T-cell activation and expansion by culture-expanded iNKT cells. (**A**) Expression of CD25 and (**B**) percentage of proliferating allogeneic CD3+ T cells when co-cultured with allogeneic DCs and increasing numbers of donor iNKT cells that were expanded for 21 days with either KRN7000, PBS44 or PBS57. Pooled data from four independent experiments. Error bars indicate standard error of the mean.

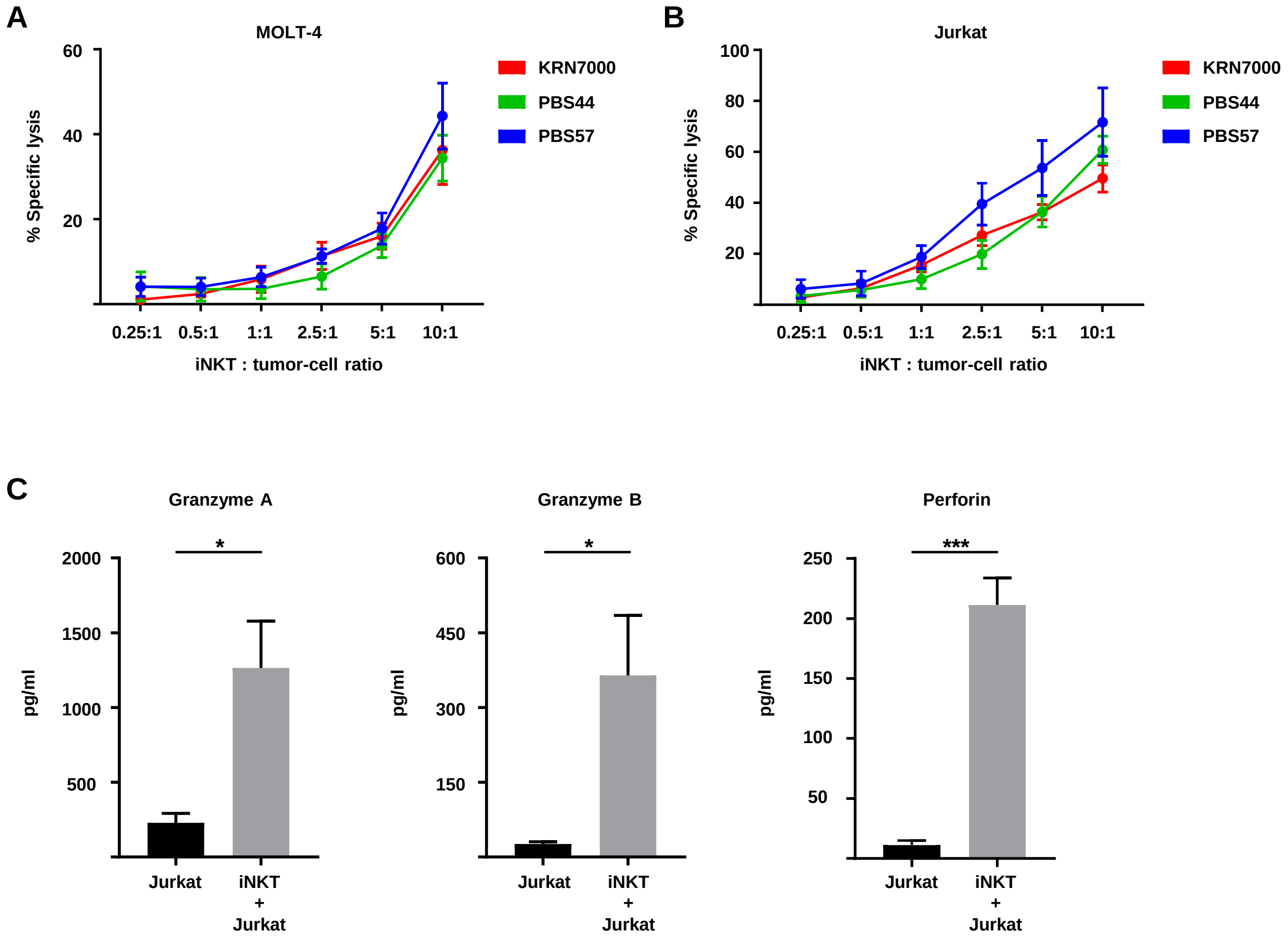

Supplemental Figure 3. Lysis of leukemia cells by culture-expanded iNKT cells. Specific lysis of (**A**) MOLT-4 and (**B**) Jurkat cells challenged with increasing numbers of iNKT cells that were expanded for 21 days with either KRN7000, PBS44 or PBS57. Each graph shows pooled data from four independent experiments with different culture-expanded iNKT cells. (**C**) Effector molecules detected in supernatants after 16 hours of co-incubation of Jurkat cells with KRN7000-expanded iNKT cells. Pooled data from three independent experiments. Error bars indicate standard error of the mean.

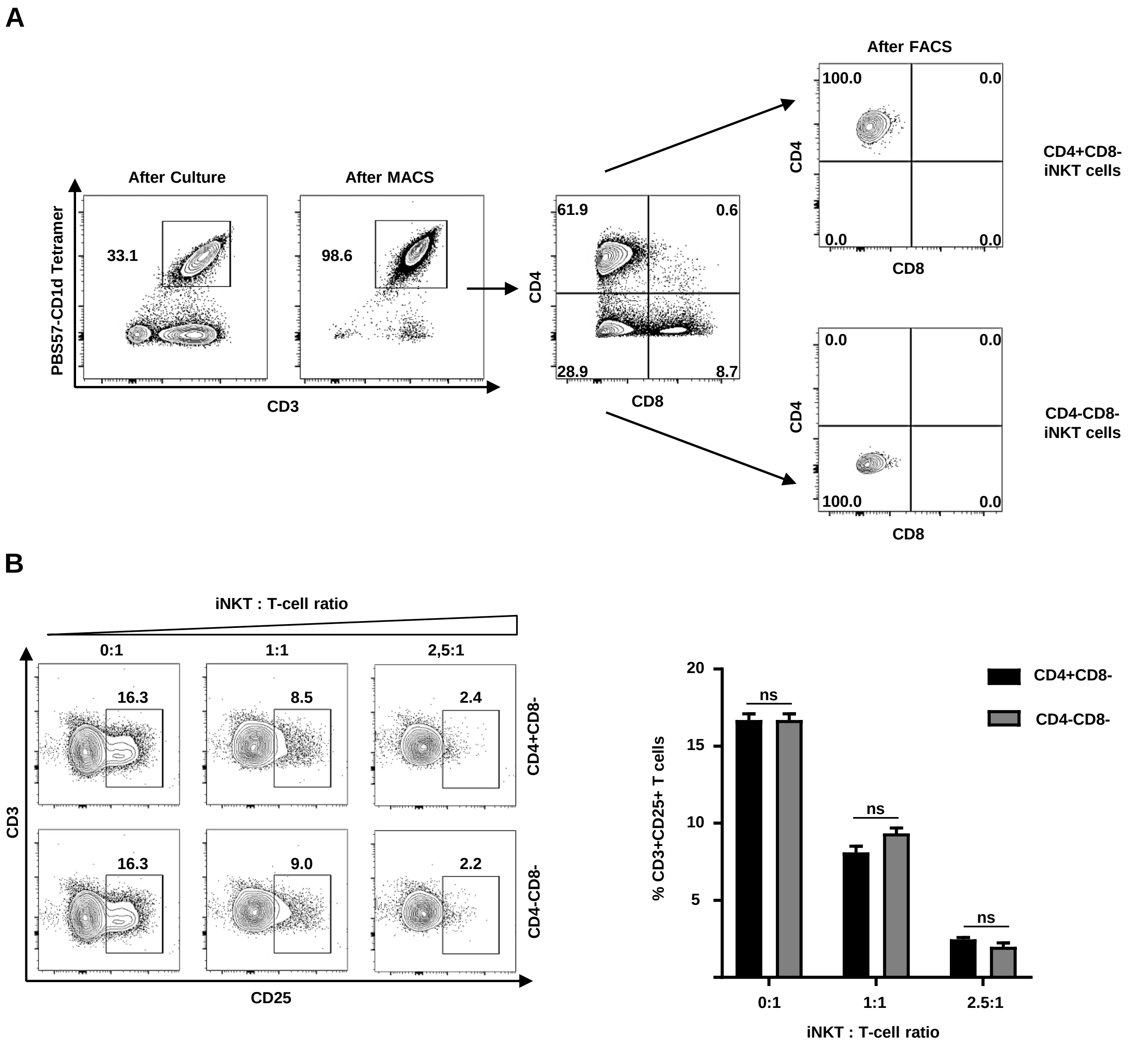

Schmid et al., Supplemental Figure 4

C

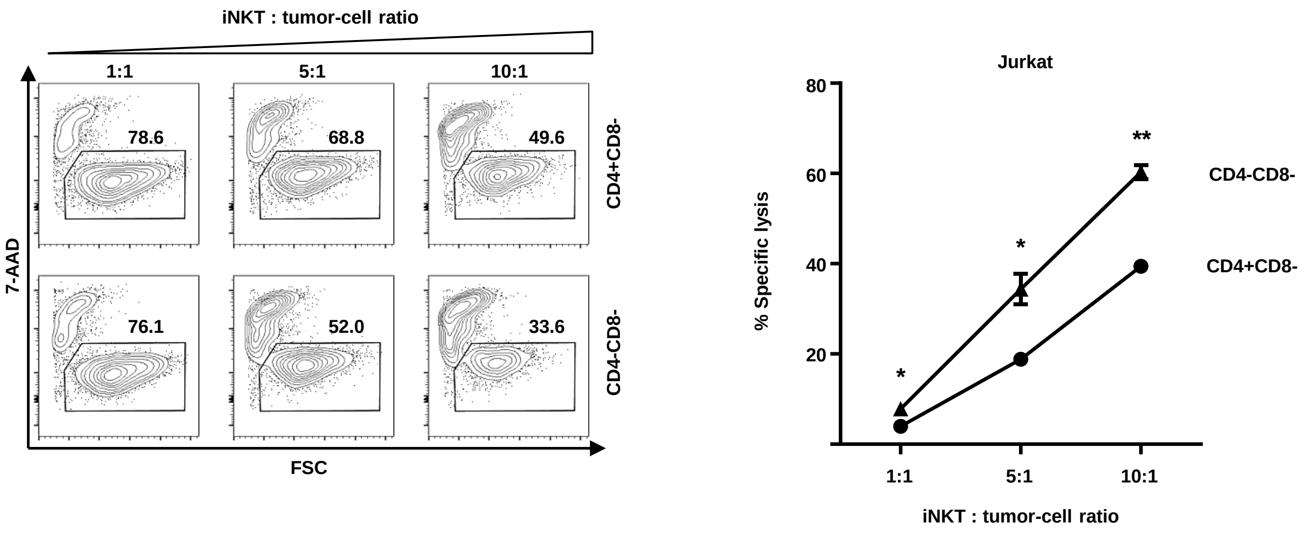

Supplemental Figure 4. Functional properties of culture-expanded iNKT-cell subsets. (**A**) Fluorescence-activated cell sorting strategy of culture-expanded human iNKT cells. (**B**) CD25 expression on live CD3+ T cells after co-incubation with CD4+CD8- and CD4-CD8- fluorescence-activated cell sorted iNKT cells. (**C**) Representative dot plots and specific lysis of Jurkat cells (82% 7-AAD- without iNKT cells) challenged with increasing numbers of CD4+CD8- and CD4-CD8- iNKT-cell subsets. All events are gated on PBS57-loaded CD1d tetramer PE negative cells to exclude iNKT cells. Shown is one representative experiment from four independent experiments. Error bars indicate standard error of the mean.
